# Allosteric Analysis of Glucocorticoid Receptor-DNA Interface Induced by Cyclic Py-Im Polyamide: A Molecular Dynamics Simulation Study

**DOI:** 10.1371/journal.pone.0035159

**Published:** 2012-04-19

**Authors:** Yaru Wang, Na Ma, Yan Wang, Guangju Chen

**Affiliations:** College of Chemistry, Beijing Normal University, Beijing, People's Republic China; Indian Institute of Science, India

## Abstract

**Background:**

It has been extensively developed in recent years that cell-permeable small molecules, such as polyamide, can be programmed to disrupt transcription factor-DNA interfaces and can silence aberrant gene expression. For example, cyclic pyrrole-imidazole polyamide that competes with glucocorticoid receptor (GR) for binding to glucocorticoid response elements could be expected to affect the DNA dependent binding by interfering with the protein-DNA interface. However, how such small molecules affect the transcription factor-DNA interfaces and gene regulatory pathways through DNA structure distortion is not fully understood so far.

**Methodology/Principal Findings:**

In the present work, we have constructed some models, especially the ternary model of polyamides+DNA+GR DNA-binding domain (GRDBD) dimer, and carried out molecular dynamics simulations and free energy calculations for them to address how polyamide molecules disrupt the GRDBD and DNA interface when polyamide and protein bind at the same sites on opposite grooves of DNA.

**Conclusions/Significance:**

We found that the cyclic polyamide binding in minor groove of DNA can induce a large structural perturbation of DNA, i.e. a >4 Å widening of the DNA minor groove and a compression of the major groove by more than 4 Å as compared with the DNA molecule in the GRDBD dimer+DNA complex. Further investigations for the ternary system of polyamides+DNA+GRDBD dimer and the binary system of allosteric DNA+GRDBD dimer revealed that the compression of DNA major groove surface causes GRDBD to move away from the DNA major groove with the initial average distance of ∼4 Å to the final average distance of ∼10 Å during 40 ns simulation course. Therefore, this study straightforward explores how small molecule targeting specific sites in the DNA minor groove disrupts the transcription factor-DNA interface in DNA major groove, and consequently modulates gene expression.

## Introduction

Allosteric modulation plays a key role in integrating and responding to multiple signals in biological systems [1]–[4]. For example, nuclear hormone receptors, such as the glucocorticoid receptor (GR) as ligand-activated transcription factor, use hormones as allosteric effectors to achieve their transcriptional regulatory activity [5], [6]. It has been proposed that DNA is a sequence-specific allosteric ligand of GR that tailors the activity of the receptor toward specific target genes [5]. The study on small-molecule modulators directed specifically to the protein-protein or protein-DNA interface has been become an area of considerable interest during the last few decades [7]–[10]. These modulators have provided useful tools for understanding gene regulatory pathways and may offer alternative approaches for modulating transcription factor activities [11]–[13]. The oversupply of transcription factors can lead to dysregulated gene expression, a characteristic of many human cancers. Cell-permeable small molecules that can be programmed to disrupt transcription factor-DNA interfaces could silence aberrant gene expression pathways and achieve the chemical control of gene networks.

More recently, pyrrole-imidazole (Py-Im) polyamides have been shown to permeate cell membranes [14], access chromatin, and interfere with protein-DNA interface [15]. Dervan and coworkers have supposed that a small polyamide molecule that competes with GR for binding to the consensus glucocorticoid response element (GRE) could be expected to disrupt GR-DNA binding specifically, and be used as a tool to identify GR target genes [11]. They showed that a cyclic Py-Im polyamide can be programmed to bind a broad repertoire of DNA sequences, and can induce allosteric modulation of DNA structure [7]. Moreover, they further proposed a hypothesis that this polyamide, as an allosteric modulator, perturbs the DNA structure in such a way that nuclear receptor binding is no longer compatible, ultimately silencing aberrant gene expression pathway [7]. The cyclic polyamide is comprised of two antiparallel ImPyPyPy strands capped at both ends by (R)-β-amino-γ-turn units. Such turn appears to reinforce an antiparallel strand alignment that prevents slippage of the amide-linked heterocyclic strands. The conformational constraints imposed by the turn and inability of the ligand to slip into a possibly more preferred orientation may impact the overall DNA structure by inducing bend and other distortions accommodated by the plasticity of DNA [7], [8]. This allosteric perturbation of the DNA helix by small molecules provides a molecular basis for the disruption of transcription factor-DNA interfaces [7]. On the other hand, it has been known that GR contains three highly conserved domains consisting of an N-terminal domain (NTD), a DNA-binding domain (DBD) and a C-terminal ligand-binding domain (LBD) [16]. Especially, since the X-ray crystal structures of GR DNA-binding domain (GRDBD) dimer binding to some genes, such as the GRDBD dimer binding to the FKBP5 gene, have been presented, it is clearly confirmed that GR modulates gene transcription via the binding of receptor dimer, especially the GRDBD dimer, to specific palindromic sequences, i.e. the glucocorticoid response elements (GREs), usually located in the cis-regulatory region of target genes [11], [17]. The GRE modulation of GR with the direct DNA-binding mechanism plays a key role in glucose metabolism, inflammation and stress [18]. Yamamoto and coworkers have proposed that the differences at the single-base-pair level of DNA were able to affect the conformation and regulatory activity of GR [5]. The crystal structure of GRDBD dimer binding to the FKBP5 gene has shown that the amino-terminal α helix in each GRDBD lies in the major groove and forms three connection sites at Lys63, Val64 and Arg68 residues with the bases of DNA [5], [19], [20]. It has been theoretically investigated for the structural and dynamical aspects of the GR activity modulation [21]–[26]. For example, Bonvin and coworkers have implemented molecular dynamics simulations on GR conformational change induced by two single point mutations and dynamical aspects of the conformational switch of GR [22]. This study further supports the allosteric regulatory activity of a transcription factor to specific target genes [22]. Moreover, there are already considerable literature precedents for the simulation researches on polyamide-DNA complexes [27]. However, the identification of allosteric perturbation of the DNA helix by cyclic polyamide, and especially how to interfere with the protein-DNA interface by the perturbation of allosteric DNA has not yet been detailed so far. Though it has been found that a polyamide molecule binds to the minor groove of a DNA sequence specifically, and GRDBD dimer binds to the major groove of DNA separately analyzed from the polyamide-DNA complex and the GRDBD dimer+DNA X-ray crystal structure, it will be expected to implement a molecular dynamics (MD) simulation study on a ternary model of polyamides+DNA+GRDBD dimer to address how polyamide molecules that bind to DNA at the regulatory regions of target genes, such as FKBP5, disrupt the GR-DNA interface, which would provide useful tools for understanding gene regulatory pathways, and for modulating transcription factor activity.

In this study, two cyclic polyamides binding to a complex of GRDBD dimer and a consensus DNA sequence from FKBP5 gene were tested in order to address how polyamides disrupt GR-DNA binding interface. We prepared four independent MD simulations, of which the first MD simulation was performed on the GRDBD dimer+DNA complex to investigate their binding properties and evaluate the accuracy of the current protocol; the second one was performed on the cyclic polyamides+DNA complex, in which the polyamides targeted to the DNA sequence 5′-WGWWCW-3′ (where W = A or T) found in the consensus GRE, to address the binding nature between cyclic polyamide and DNA; the third one was performed on the polyamides+DNA+GRDBD dimer complex to investigate the binding inhibition of GRDBD dimer to DNA molecule induced by polyamides; the last one was performed on an allosteric DNA+GRDBD dimer complex. In the simulation of this model, the allosteric DNA backbone was restrained to investigate how allosteric DNA conformation affects the GR protein and DNA interface.

## Materials and Methods

### Initial structures

**Figure 1:**
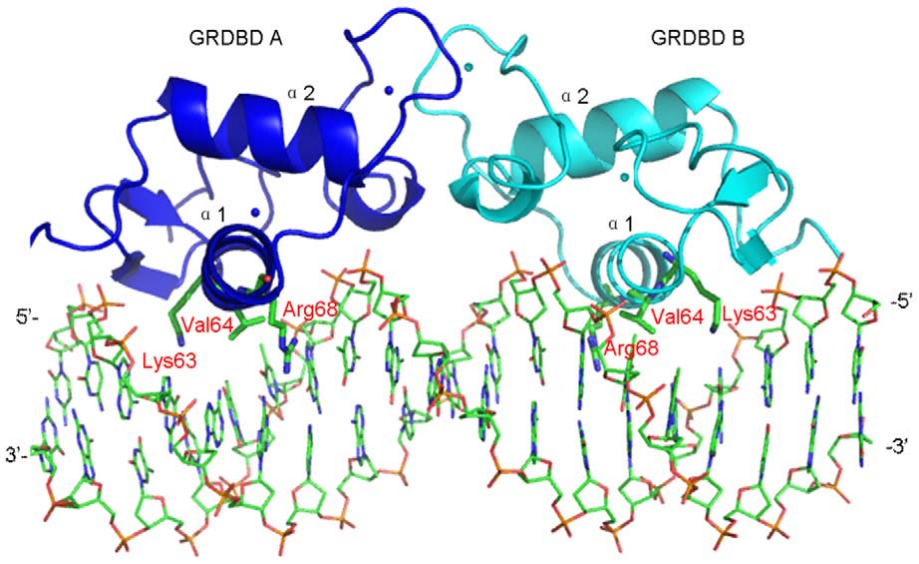
Structure of GRDBD dimer bound to DNA. The structure of a GR DNA-binding domain (GRDBD) dimer bound to the 19 bp DNA fragment along with the connection sites of GRDBD. The GRDBD dimer has been shown by GRDBD A (blue) on the left and GRDBD B (cyan) on the right respectively with α1 and α2 helices.

**Figure 2:**
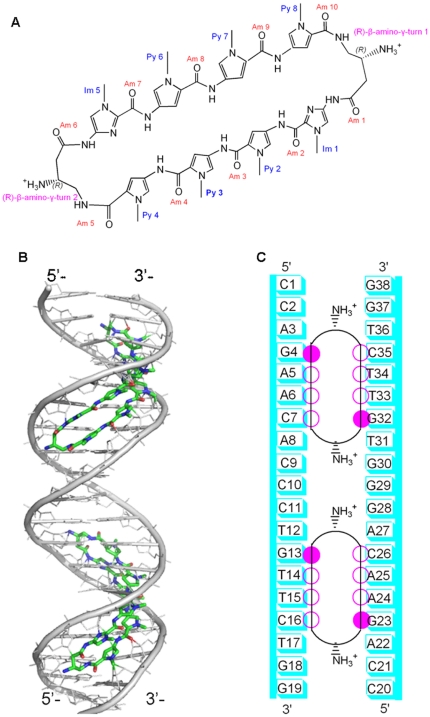
Sketches of polyamide and polyamide+DNA with the connection sites. (A) Component sketches of cyclic polyamide; (B) Initial structure of cyclic polyamides-DNA system (Poly+DNA); (C) The connection sites between cyclic polyamides and DNA presented with closed circles designating N-methylimidazole, open circles designating N-methylpyrrole, and arc-lines substituted with ammoniums designating the β-amino-substituted γ-turn unit.

On the basis of previous experimental and MD simulation studies of GRDBD dimer+DNA complexes [5], [22], [28], the initial structure of GRDBD dimer+DNA complex (assigned as GRDBD+DNA model) as the starting structure for the MD simulation was taken from the X-ray crystal structure (PDB entry 3G6P) [5]. The GRDBD+DNA model consists of a 19 bp palindromic DNA fragment, 5′-d(CCAGAACACCCTGTTCTGG)-3′, and a GRDBD dimer in which each GRDBD is assigned as GRDBD A or GRDBD B containing α1 and α2 helices (shown in Figure 1). Only α1 helix in a GRDBD contacts to the bases of DNA. In order to choose an initial conformation of two polyamide molecules binding to DNA molecule for MD simulations, the DNA coordinate was taken from the GRDBD+DNA X-ray crystal structure (PDB entry 3G6P) [5], and the two cyclic polyamide coordinates were taken from the polyamide-DNA X-ray crystal structure (PDB entry 3OMJ) [7]. The two cyclic polyamides were docked into the minor groove of DNA duplex by using AutoDock 4.0 [29], [30] with an AutoDockTool [31] (assigned as Poly+DNA model and shown in Figure 2). Details of the docking procedure are available in Text S1. In order to investigate an influence of DNA bound by polyamide molecules on combination of GR protein, a GRDBD dimer has been docked manually to DNA major groove of the Poly+DNA model that was taken from an average structure of simulation for the Poly+DNA model to build a Poly+DNA+GRDBD model. The docked positions and distances between GRDBD and DNA molecule in the Poly+DNA+GRDBD model were referenced from the X-ray crystal structure of the GRDBD+DNA model [5]. The alloDNA+GRDBD model was built by manually docking the GRDBD dimer to the major groove of allosteric DNA, the coordinate of which was taken from the time-averaged structure of simulated Poly+DNA model with the same 19 bp palindromic DNA sequence in the GRDBD+DNA model. Four different initial structures with the GRDBD dimer docked around the connection sites of GRDBD dimer and DNA molecule for each of the Poly+DNA+GRDBD and alloDNA+GRDBD models were chosen as the starting structures for the MD simulations. This provides a valid test of whether those MD simulations are capable of driving significantly distinct starting structures to a non-distinguishable one through the course of each simulation. To compare the differences of conformations between the disturbed DNA molecule and a canonical DNA molecule, a canonical B-DNA molecule simulation was also performed. Given that each strand of DNA has some phosphate groups, 56 Na^+^ and 16 Cl^−^ counterions for the GRDBD+DNA model are added to achieve electroneutrality and to satisfy the experimental ionic strength, namely 100 mM for the GRDBD dimer+DNA complex [5]. Similar counterion processes are applied to the Poly+DNA, Poly+DNA+GRDBD and alloDNA+GRDBD models. The systems were explicitly solvated by using the TIP3P water potential inside a rectangular box large enough to ensure the solvent shell extended to 12 Å in all directions.

### Force field parameter preparation

The atom types for the studied polyamide were generated by using the ANTECHAMBER module in AMBER9 program [32]. The force field parameters and RESP charges of the zinc centers of the GRDBD dimer were referenced from the previous work [33].

### Molecular dynamics simulation protocols

All MD simulations were carried out using the AMBER9 package [32] with a classical AMBER parm99 [34], [35] together with the parmbsc0 refinement [36] and gaff [37] force field parameters. Details of the MD protocols are given in Text S2. The summaries of the simulations performed in this work have been also given in Table S1.

### Free-energy analyses

The molecular mechanics Poisson–Boltzmann surface area (MM-PBSA) method [38]–[41] in AMBER9 package was employed to perform the free energy analyses. The binding free energy was computed through calculating the free energy differences of ligand, receptor and their complex as follows:
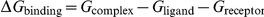
In MM-PBSA, the free energy (*G*) of each state is estimated from molecular mechanical energy *E*_MM_, solvation free energy *G*_SOLV_ and vibrational, rotational, and translational entropies *S*, respectively.
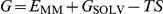


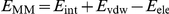


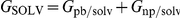
where *T* is the temperature; *E*_int_ is internal energy, i.e. the sum of bond, angle, and dihedral energies; *E*_vdw_ is van der Waals energy; *E*_ele_ is electrostatic energy; *G*_SOLV_ is the sum of electrostatic solvation free energy, *G*_pb/solv_, and the non-polar salvation free energy, *G*_np/solv_. The entropy *S* is estimated by a normal mode analysis of the harmonic vibrational frequencies, calculated using the Nmode module in Amber9 package [42], [43]. Prior to the normal mode calculations, each structure was fully minimized using a distance dependent dielectric of ε = 4r (r is the distance between two atoms) to mimic the solvent dielectric change from the solute to solvent until the root-mean-square of the elements of the gradient vector was less than 5×10^−4^ kcal·mol^−1^·Å^−1^. Then, the entropy was calculated based on standard statistical mechanics expressions [39], [44]. Computational details are available in Text S3.

### DNA groove parameter analyses

The frequency distributions (fraction of the time spent in each conformation) from the trajectories of simulations for the models and a canonical B-DNA were calculated using the CURVES program [45] to investigate the distortion of DNA. To account for the distortion of the whole DNA backbone, the overall bend, tilt and roll angles of the DNA time-averaged structures for the studied models were calculated from the CURVES outputs using MadBend program [46]. Details of the calculation method are available in Text S4.

## Results

**Figure 3:**
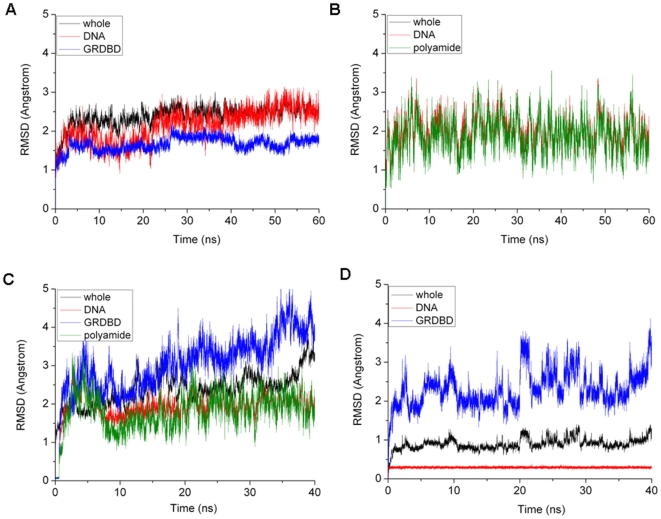
RMSD values of GRDBD+DNA, Poly+DNA, Poly+DNA+GRDBD, and alloDNA+GRDBD models. RMSD values of all backbone atoms of the whole system (black), GRDBD dimer (blue), DNA alone (red) and polyamides alone (olive) with respect to the corresponding starting structure in the simulations of (A) GRDBD+DNA, (B) Poly+DNA, (C) Poly+DNA+GRDBD, and (D) alloDNA+GRDBD.

The root-mean-square deviation (RMSD) values of all backbone atoms referenced to the corresponding starting structures over two trajectories for the GRDBD+DNA and Poly+DNA models were examined to determine if each system had attained equilibrium. It is often considered that small RMSD values of one simulation indicate a stable state of the system. However, the large RMSD values during a course of simulation suggest large conformation changes of investigated system. Plots of RMSDs of the four system simulations over time are shown in Figure 3. It can be seen from Figures 3A and B that the GRDBD+DNA and Poly+DNA systems reached equilibrium after 40 ns, and their energies were found to be stable during the remainder of each simulation. Therefore, the trajectory analysis for the two systems has extracted the equilibrated conformations between 40 ns and 60 ns of simulation time, recording 10000 snapshots at every 2 ps time-interval of each trajectory. The RMSD variation analysis for the Poly+DNA+GRDBD and alloDNA+GRDBD models is shown in Figures 3C and D, respectively. It can be seen that the RMSD values of the GRDBD dimer at the connection positions for each of these two systems occur significant changes, which predicts that the GRDBD dimer moves away from the original positions in the initial structure of the Poly+DNA+GRDBD or alloDNA+GRDBD model. Only the last 500 snapshots (1 ns) of each trajectory of the two systems are used for the final changed structure analysis due to the allosteric simulations for the two models.

### The calculated data of free energies favor the binding of polyamides to DNA over that of GRDBD dimer to DNA

Since the competitive binding of polyamide molecule and GR protein to the consensus GRE, the binding free energies were investigated by using the MM-PBSA methodology with the single-trajectory analysis for the GRDBD+DNA and Poly+DNA+GRDBD models. The binding free energy for the Poly+DNA model was calculated by using the same method with the triplet-trajectory analysis due to the certain changes of DNA conformation caused by the polyamide binding. Table 1 gives all energy terms and the total binding free energies for these systems. Our results indicate that the Poly+DNA model is energetically favorable with the binding free energy of −95.39 kcal·mol^−1^ over the GRDBD+DNA model with that of −5.14 kcal·mol^−1^ which is consistent with the experimental result of −8.50 kcal·mol^−1^ analyzed by the dissociation constant K_d_ [5]. Therefore, the DNA binding of polyamide prior to GR protein is possible when the polyamide accesses certain chromatins through permeating cell membranes. Furthermore, the calculated average binding free energy of 12.73 kcal·mol^−1^ for the GRDBD dimer binding to DNA molecule attached by two polyamides in the Poly+DNA+GRDBD model predicts the instabilities of GR protein combining with the polyamides+DNA complex.

**Table 1:** MM-PBSA free energy (kcal·mol^−1^) components for the GRDBD+DNA, Poly+DNA, Poly+DNA+GRDBD, and alloDNA+GRDBD models.

	GRDBD+DNA	Poly+DNA	Poly+DNA+GRDBD	alloDNA+GRDBD
Receptor	DNA	DNA	DNA and polyamides	DNA
Ligand	GRDBD dimer	polyamides	GRDBD dimer	GRDBD dimer
ΔE_ele_	−975.65	−1635.28	−1031.86	−943.23
ΔE_vdw_	−124.49	−233.91	−62.74	−65.49
Δ*E*_int_	0.00	0.60	0.00	0.00
ΔG_np/solv_	−22.91	−24.51	−12.90	−12.79
ΔG_pb/solv_	1038.75	1711.29	1018.15	964.03
ΔG_np_	−147.27	−258.42	−75.64	−78.28
ΔG_pb_	63.10	76.01	−13.71	20.80
ΔTS	−79.16	−86.42	−74.66	−83.64
ΔH_binding_	−84.30	−181.81	−61.93	−57.48
ΔG_binding_	−5.14(−8.50^a^)	−95.39	12.73	26.16

a Is taken from Ref. [5].

### The DNA distortion caused by polyamide molecules inhibits the binding of GRDBD dimer to DNA

#### 1. Variations of DNA groove parameters

**Figure 4:**
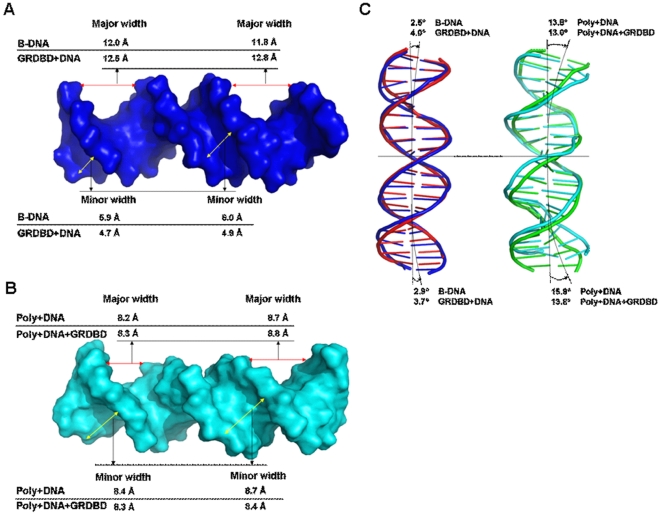
DNA groove parameters of B-DNA, GRDBD+DNA, Poly+DNA and Poly+DNA+GRDBD models. The minor and major groove widths for the time-average structures of the DNA conformations for (A) B-DNA and GRDBD+DNA, and (B) Poly+DNA and Poly+DNA+GRDBD; (C) The bend angles of the DNA conformations for B-DNA, GRDBD+DNA, Poly+DNA and Poly+DNA+GRDBD models.

Figure 4 shows the DNA groove parameters around the connection sites, G4:C35, A5:T34, A6:T33, C7:G32, G13:C26, T14:A25, T15:A24 and C16:G23 base pairs (see Figure 2C) of DNA molecule for the B-DNA, GRDBD+DNA, Poly+DNA and Poly+DNA+GRDBD models. The DNA groove structure variations caused by the GRDBD dimer and polyamides are much different upon the major groove binding mode of GR protein and the minor groove binding mode of polyamide molecule [5], [11]. The calculated DNA average minor groove width, major groove width and bend angle toward DNA major groove of 6.0 Å, 11.9 Å and 2.7°, respectively, for the simulated B-DNA molecule are close to those of 5.8 Å, 11.7 Å and <4° for the canonical B-DNA [47], [48]. It can be seen from Figure 4 that GRDBD binding to DNA does not cause significant DNA groove parameter variations with the corresponding data of 4.8 Å, 12.7 Å and 3.9°. However, the polyamide binding to DNA in the Poly+DNA model causes larger variations in DNA groove parameters with the corresponding data of 8.6 Å, 8.5 Å and ∼14.9°. Similar results have been found in the Poly+DNA+GRDBD model (see Figure 4), which indicates that the DNA distortion caused by the polyamide binding is not changed by the further combination of GRDBD. Furthermore, the changes of DNA groove parameters along with the base pairs have been analyzed in Figure S1. It can be seen that the groove parameter variations for the Poly+DNA and Poly+DNA+GRDBD models occur mainly at the connection sites of the GRDBD dimer-DNA and polyamides-DNA interfaces. These results predict the increases of DNA minor groove width and major groove depth with the decreases of the minor groove depth and major groove width at the interaction regions in the Poly+DNA and Poly+DNA+GRDBD models.

#### 2. Dynamics analysis of DNA base pairs

The DNA base-pair helical analysis can evaluate the characteristics of DNA conformation and curvature. The frequency distributions of DNA helical parameters for the B-DNA, Poly+DNA and Poly+DNA+GRDBD models have been shown in Figure S2 and Figure S3. The opening parameters for the DNA molecule in the Poly+DNA and Poly+DNA+GRDBD models at the connection sites, such as G4:C35, A5:T34, A6:T33, C7:G32, G13:C26, T14:A25, T15:A24 and C16:G23 base pairs, are changed by −10°∼−15° away from the B-DNA molecule, which results in a widening of minor groove and a compression of major groove due to the base pairs opening toward the DNA major groove. The roll parameters for the DNA molecule in the Poly+DNA and Poly+DNA+GRDBD models at the connection sites, A3·G4, G4·A5, and G32·T31 base-pair steps, are changed by 20°, −10° and 30°, respectively, away from the B-DNA molecule, which contributes to the significant bend of the DNA helix toward the major groove. Furthermore, the twist parameter changes of the two systems at the A3·G4, G4·A5 and G32·T31 base-pair steps with the corresponding values of −5°, 10° and −20°, respectively, were observed due to the anticorrelation of roll and twist parameters.

#### 3. Binding property change of GRDBD dimer to a normal-DNA/distorted-DNA molecule

**Figure 5:**
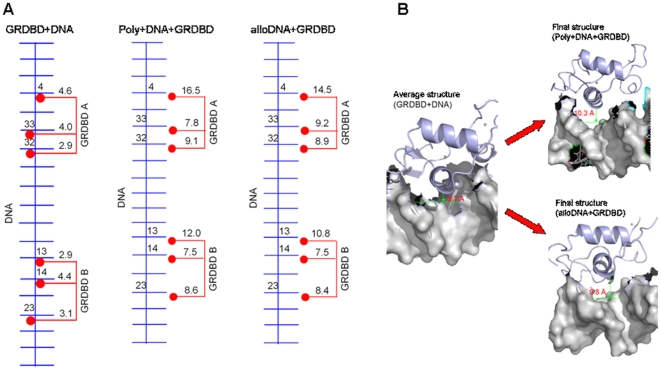
Distances and structures for GRDBD+DNA, Poly+DNA+GRDBD and alloDNA+GRDBD models. (A) The distances of connection sites between the residues of GRDBD dimer and bases of DNA for GRDBD+DNA, Poly+DNA+GRDBD and alloDNA+GRDBD models; (B) The time-average structure of the GRDBD+DNA model, the final structure of the Poly+DNA+GRDBD model and the final structure of the alloDNA+GRDBD model in the simulations along with the average distances of the connection sites between the two α1 helices of GRDBD dimer and DNA molecule.

It has been found from the allosteric simulation for the Poly+DNA+GRDBD model that the distorted DNA molecule caused by the polyamide combination is unfavorable to combine with GR protein. The average distance of the connection sites between the two α1 helices of GRDBD dimer and DNA-combined by polyamide molecules from the initial average value of 3.8 Å changes to the final average value of 10.3 Å with a standard deviation of 0.8 Å through 40 ns simulation of the Poly+DNA+GRDBD model (see Figure 5A). The GRDBD dimer moved away from the DNA major groove in the later period of simulation for the Poly+DNA+GRDBD model (see Figure 5B), which indicates a decrease of interaction of the GR protein to DNA molecule, possibly affecting the GR modulation function to DNA transformation. The percentages of occurrences of hydrogen bonds in the MD simulations, and the decompositions of ΔE_vdw_ for the complexes into residue components have been shown in Table S2 and Figure S4, respectively, for the GRDBD+DNA and Poly+DNA+GRDBD models. The hydrogen bond occupancies for the GRDBD+DNA model occur apparently in the connection sites, the Lys63 and Arg68 residues of GRDBD and G4:C35 and C7:G32 base pairs of DNA, which supports the previous experimental observations (see Figure S5) [5]. As expected, the original hydrogen bonds and van der Waals contacts between the GRDBD dimer and DNA molecule in the GRDBD+DNA model disappear in the Poly+DNA+GRDBD model.

### The DNA structure distortion plays a key role in inhibition of GRDBD dimer binding to DNA

It has been found from the allosteric simulation of the alloDNA+GRDBD model in which the distorted DNA conformation was restrained, that the distorted DNA molecule without any polyamide combination is also unfavorable to combine with GR protein. Similar to the Poly+DNA+GRDBD model, the average distance of the connection sites between the two α1 helices of GRDBD dimer and the distorted-DNA changes from the initial average value of 4.0 Å to the final average value of 9.8 Å with a standard deviation of 0.7 Å through 40 ns simulation of the alloDNA+GRDBD model (see Figure 5A). The GRDBD dimer also moved away from the DNA major groove in the later period of simulation for the alloDNA+GRDBD model (see Figure 5B), which indicates the decrease of interaction between the GRDBD dimer and distorted DNA, and the effect of DNA molecule distortion on inhibiting the combination of GR protein with DNA. Moreover, the binding free energy for the GRDBD dimer binding to distorted DNA in the alloDNA+GRDBD model is 26.16 kcal·mol^−1^ (see Table 1), which indicates further that the GR protein binding to the allosteric DNA molecule is energetically unfavorable.

## Discussion

### DNA structure perturbation induced by polyamide

Generally, the pyrrole-imidazole (Py-Im) polyamides bind in the minor groove of DNA sequence specifically [15], [49], encoded by side-by-side arrangements of N-methylpyrrole (Py) and N-methylimidazole (Im) carboxamide monomers. Im-Py pairs distinguish G:C from C:G base pairs, whereas Py-Py pairs are degenerate for T:A and A:T base pairs [15], [50]. Especially, antiparallel ImPyPyPy strands are connected by (R)-β-amino-γ-turn units to create cyclic-shaped polyamide. It can be seen from the Poly+DNA and Poly+DNA+GRDBD models, the cycle ImPyPyPy polyamide is capable of binding specifically in the minor groove of DNA sequence with affinity and selection comparable to natural DNA-binding proteins [15]. The aromatic amino acids in cycle polyamide are bound with an N-to-C orientation of each strand of the cycle polyamide adjacent to the 5′-3′ direction of the DNA in the Poly+DNA and Poly+DNA+GRDBD models, which results in the geometry distortion of DNA (see Figure 4).

The occupancies of all possible hydrogen bonds between the polyamides and DNA bases in the Poly+DNA and Poly+DNA+GRDBD models were identified by calculating the percentages of occurrences of hydrogen bonds in the MD simulations, and the obtained data of hydrogen bond occupancies and H-bond map are shown in Figure S6. The occupancies of observed hydrogen bonds between the polyamides and DNA bases in both models are similar because the GRDBD dimer binding to the DNA major groove does not yet significantly affect the polyamide and DNA interfaces. The hydrogen bond formations at the connection sites of polyamide and DNA interface cause the dynamics changes of DNA bases and the DNA groove distortion. For example, for the upper polyamide and DNA interface, the hydrogen bonds between the N-H group of amide (Am) 4 of polyamide and O2 atom of C7 base, between the N atom of Im 5 of polyamide and N-H group of G32 base (pairing to C7 base) and between the N-H group of Am 6 of polyamide and the N3 atom of G32 base are maintained with the occupancies of ∼94%, ∼94% and ∼99% of simulation times, respectively, in the Poly+DNA and Poly+DNA+GRDBD models (see Figure 2 and Figure S6). The hydrogen bonds between the N-H group of (R)-β-amino-γ-turn 2 in the polyamide and O2 atom of T31 base, and between the N-H group of Am 7 and O2 atom of T33 base are also maintained with the occupancies of ∼96% and ∼98% of simulation times, respectively. These hydrogen bonds may mainly result in the opening and buckling of C7:G32 base pair with the corresponding changed values of ∼−10° and ∼20°, respectively, the rolling and twist of G32·T31 base-pair step with the changed values of ∼30° and ∼−20°, respectively, and the shifts of both G32·T31 and G32·T33 base-pair steps with the changed values of ∼1 Å compared with the B-DNA molecule (see Figure S2 and Figure S3). Similar results can be found at other connection sites in the Poly+DNA and Poly+DNA+GRDBD models. Moreover, the hydrogen bond formations between the (R)-β-amino-γ-turn and the bases of DNA are due to a conformational inversion where the β-carbon conformational preference is puckered up and away from the DNA minor-groove floor, aligning the β-ammonium substituent along the groove floor. This may provide a structural basis for the DNA base-pair sequence specificity of the β-amino-γ-turn recognition unit, which is consistent with the experimental observations and may possess more favorable DNA-binding affinities [7], [11]. It is clearly demonstrated by this work that the two polyamides strongly perturb the overall DNA helix structure, resulting in the widening of minor groove of DNA up to ∼8 Å while simultaneously the compression of the major groove by 4 Å, and the DNA helix bend of ∼15° toward the major groove. Summarily, these computational results indicate that the polyamide binds to DNA with a fairly high affinity accompanying with DNA structure perturbation, which is consistent with the discussion in the section on Results.

### GRDBD dimer moves away from the DNA major groove

**Figure 6:**
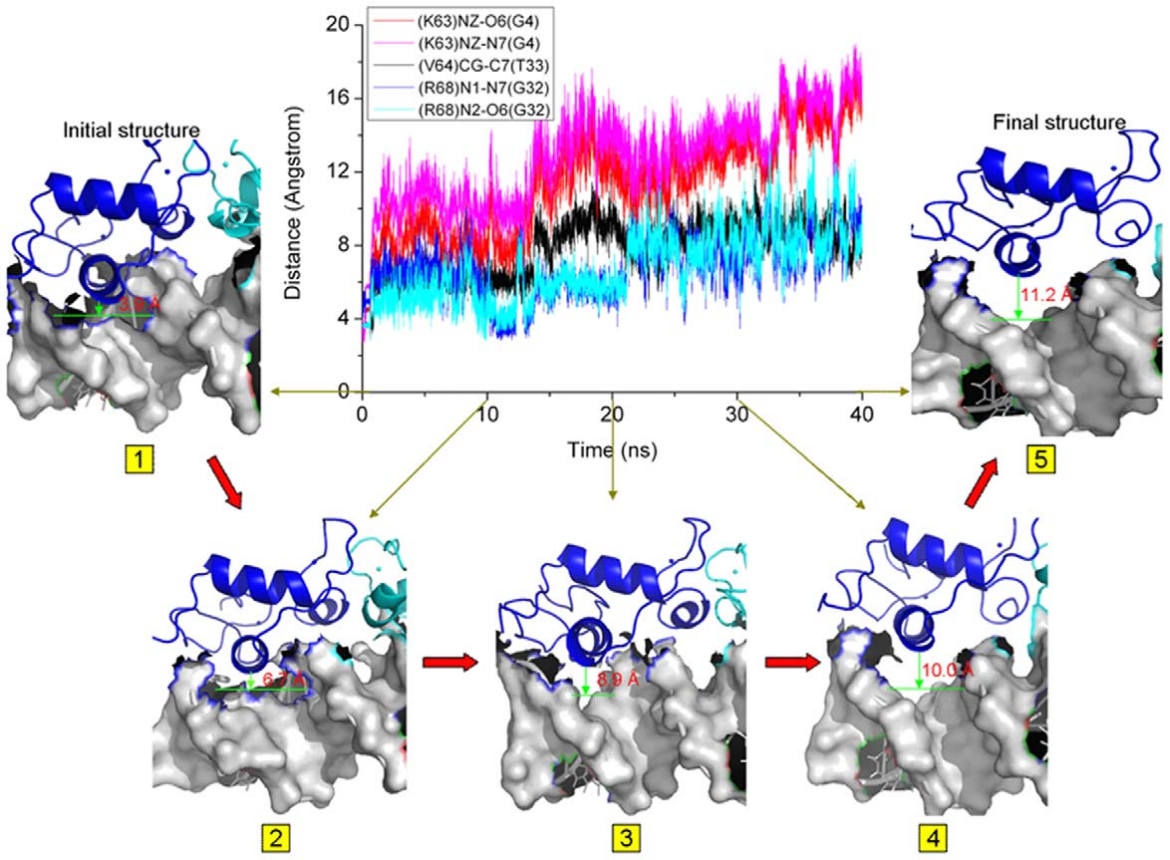
Distances at certain times for Poly+DNA+GRDBD model. The center image is the time-dependence of distances at the connection sites between the residues of GRDBD A and the bases of DNA during the simulation of the Poly+DNA+GRDBD model. Around the center image, five snapshots are extracted from the Poly+DNA+GRDBD trajectory at the times of 0 (1), 10 (2), 20 (3), 30 (4) and 40 (5) ns, respectively, along with the average distances of connection sites.

It has been found from the course of simulation for the Poly+DNA+GRDBD model that the GRDBD region moves away from the DNA major groove along with the conformational change of DNA induced by the polyamides. The α1 helix in GRDBD A contains Lys63, Val64 and Arg68 residues as interaction region with the bases of DNA, so care must be taken in analyzing the interactions at these connection sites. There are gross changes in the distances between Lys63, Val64 and Arg68 residues of GRDBD A and G4, T33 (in A6:T33 base pair), G32 (in C7:G32 base pair) bases of DNA molecule, respectively, during the simulation of Poly+DNA+GRDBD model. The corresponding distance values for GRDBD A were shown in Figure 6. It can be seen that the conformational changes of GRDBD A and DNA interface at times of 0 ns, 10 ns, 20 ns, 30 ns and 40 ns during the simulation of Poly+DNA+GRDBD model correspond to α1 helix of GRDBD A moving out gradually from the DNA major groove with the average distances from 3.9 Å, 6.7 Å, 8.9 Å, 10.0 Å to 11.2 Å. These observations result from the destroying of the original hydrogen bonding net work at the GRDBD and DNA interface caused by the distortion of DNA, involving the opening, rolling, etc., of the connected DNA bases. Such distortion of DNA is induced by the polyamide binding. For example, the original hydrogen bonds between two N-H groups of Arg68 residue of GRDBD A and O6, N7 atoms of G32 base in the GRDBD+DNA model with the occupancies of ∼97% and ∼98% of simulation time, respectively, have been destroyed in the Poly+DNA+GRDBD model due to the opening, buckling, rolling, twist, and shifts of the C7:G32 base pair, G32·T31 and G32·T33 base-pair steps discussed above. Moreover, the van der Waals contact between the Val64 residue and T33 base (in A6:T33 base pair) decreases considerably due to the helical parameter changes of A6:T33 base pair (see Figure S4). Since these contacts are missed during the simulation, it is possible that a movement of GRDBD out from the DNA major groove occurs in the later period of simulation for the Poly+DNA+GRDBD model. For the GRDBD B and DNA interface, similar results can be observed due to the symmetry properties of GRDBD dimer and the typically palindromic DNA sequence. Similar results for the alloDNA+GRDBD model have been obtained and shown in Figure S7. Consequently, it can be seen from the data analysis above that the DNA shape, especially the groove width, plays a critical role in the transcription factor binding.

### Polyamide has been used successfully to perturb gene transcription

Specific protein-DNA interactions are the interfaces where information from protein signaling is converted into programs of gene expression. Used in the context of simulation analysis, small molecules that perturb this interface in a predictable manner could become useful tools for regulating gene expression [11]. Programmable DNA-binding Py-Im polyamides studied by Dervan and coworkers offer a chemical approach for perturbing protein-DNA interactions [7]. The present study on the distortion of the GR-DNA interface induced by the binding of cyclic polyamide through the simulation of the ternary system, Poly+DNA+GRDBD model, offers additional perspective in the mechanistic study of GR gene regulatory action and its dual transactivation/transrepression mechanisms. The further investigation of the alloDNA+GRDBD model predicts that GR gene regulatory action can be perturbed by an allosteric DNA molecule induced by any perturbing factors due to the destroying of GR and allosteric DNA interface during the model simulation, which reveals the important of significant modulation of DNA shape for mechanistic studies of GR-modulated gene expression.

It is reported that although the core GR binding sequences (i.e. 15 base pairs) across the set of GREs varied extensively around a consensus, the sequence at an individual GRE was conserved with 5′-WGWACW-3′ (W = A, T, G or C) sequence [51]. Therefore, it may be suggested that the cyclic polyamides binding to DNA may affect most GREs due to the specific DNA sequence of 5′-WGWWCW-3′ (W = A or T) bound by a cyclic polyamide [7]. Reorienting the α1 helix of GRDBD at the GRDBD-DNA interface through mutating the residues of GRDBD may provide a useful tool to counteract inhibition of GR binding to DNA induced by polyamides [12], [52], [53].

### Conclusions

A series of molecular dynamics simulations demonstrated that the combination of polyamides with the consensus DNA sequence are more energetically favorable than that of the transcription factor GR; and that the DNA distortion inhibits the interaction of the GR protein to DNA molecule. These results demonstrate that sequence-specific minor-groove ligands could modulate DNA conformation which consequently influences the protein-DNA binding at the major groove region. Namely, the narrow and deep perturbed surface of DNA major groove upon polyamide binding is too small to accommodate the width of recognition domain with a standard α-helix in the transcription factor GR. These simulations also explored how polyamide binding in the minor groove of DNA made the GR DNA-binding domain move gradually away from the DNA major groove along with the compression of the DNA major groove. This study also provides strong evidence that the narrow major groove surface of allosteric DNA without polyamide binding becomes incompatible with GR protein-DNA binding. These results will be helpful to understand the mechanism of allosteric DNA control over transcription factor regulatory networks by small molecules binding at distinct locations on promoters of selected genes.

## Supporting Information

Figure S1 **Groove widths and depths of B-DNA, GRDBD+DNA, Poly+DNA and Poly+DNA+GRDBD models.** Minor groove widths (A), minor groove depths (B), major groove widths (C) and major groove depths (D) for the time-averaged structures of the DNA conformations in B-DNA (red line with diamond), GRDBD+DNA (blue line with square), Poly+DNA (cyan line with circle), and Poly+DNA+GRDBD (green line with up-triangle) models.
(TIF)

Figure S2 **Frequency distributions of DNA base-pair helical parameters of B-DNA, GRDBD+DNA, Poly+DNA and Poly+DNA+GRDBD models.** Selected frequency distributions of the representative DNA duplex base-pair helical parameters for the central binding base-pairs for B-DNA (red line), GRDBD+DNA (blue line), Poly+DNA (cyan line), and Poly+DNA+GRDBD (green line) models.
(TIF)

Figure S3 **Frequency distributions of DNA base-pair step parameters of B-DNA, GRDBD+DNA, Poly+DNA and Poly+DNA+GRDBD models.** Selected frequency distributions of the representative DNA duplex base-pair step parameters for the central binding base-pairs for B-DNA (red line), GRDBD+DNA (blue line), Poly+DNA (cyan line), and Poly+DNA+GRDBD (green line) models.
(TIF)

Figure S4 **Energy decompositions of GRDBD dimer.** MM-PBSA energy decompositions presented in the two α1 helical regions for the GRDBD dimer in kcal·mol^−1^: blue bars for the GRDBD+DNA model; cyan bars for the Poly+DNA+GRDBD model; green bars for the alloDNA+GRDBD model.
(TIF)

Figure S5 **Contact sites and distances of GRDBD dimer with DNA.** Diagrams of the calculated key contact sites and the corresponding distances (Å) of the GRDBD dimer with DNA for the simulated GRDBD+DNA model (left) compared with the experimental results (right). Hydrogen bond interactions indicated by black arrows and van der Waals interactions indicated by black line.
(TIF)

Figure S6 **Hydrogen bonds between polyamides and DNA.** Hydrogen bond map for two polyamides in the Poly+DNA and Poly+DNA+GRDBD models.
(TIF)

Figure S7 **Distances at certain times for alloDNA+GRDBD model.** The center image is the time-dependence of distances at the connection sites between the residues of GRDBD A and the bases of DNA during the simulation of the alloDNA+GRDBD model. Around the center image, five snapshots are extracted from the alloDNA+GRDBD trajectory at the times of 0 (1), 10 (2), 20 (3), 30 (4) and 40 (5) ns, respectively, along with the average distances of connection sites.
(TIF)

Table S1 **Summary of the simulations performed in this work.**
(TIF)

Table S2 **The occupancy (%) of hydrogen bonds between the GRDBD dimer and DNA for the GRDBD+DNA, Poly+DNA+GRDBD, and alloDNA+GRDBD models.**
(TIF)

Text S1 **Docking protocols for Poly+DNA model.**
(DOC)

Text S2 **Molecular dynamics simulation protocols used in this work.**
(DOC)

Text S3 **MM-PBSA calculation for free energy.**
(DOC)

Text S4 **DNA helical parameter analysis of trajectories.**
(DOC)

## References

[1] van Tilborg PJA, Czisch M, Mulder FAA, Folkers GE, Bonvin AMJJ (2000). Changes in dynamical behavior of the retinoid X receptor DNA-binding domain upon binding to a 14 base-pair DNA half site.. Biochemistry.

[2] Álvarez LD, Martí MA, Veleiro AS, Misico RI, Estrin DA (2008). Hemisuccinate of 21-hydroxy-6,19-epoxyprogesterone: A tissue-specific modulator of the glucocorticoid receptor.. Chem Med Chem.

[3] Green SM, Coyne HJ, McIntosh LP, Graves BJ (2010). DNA binding by the ETS protein TEL (ETV6) is regulated by autoinhibition and self-association.. J Biol Chem.

[4] Pufall MA, Lee GM, Nelson ML, Kang HS, Velyvis A (2005). Variable control of ets-1 DNA binding by multiple phosphates in an unstructured region.. Science.

[5] Meijsing SH, Pufall MA, So AY, Bates DL, Chen L (2009). DNA binding site sequence directs glucocorticoid receptor structure and activity.. Science.

[6] Álvarez LD, Martí MA, Veleiro AS, Presman DM, Estrin DA (2008). Exploring the molecular basis of action of the passive antiglucocorticoid 21-hydroxy-6,19-epoxyprogesterone.. J Med Chem.

[7] Chenoweth DM, Dervan PB (2010). Structural basis for cyclic Py-Im polyamide allosteric inhibition of nuclear receptor binding.. J Am Chem Soc.

[8] Chenoweth DM, Dervan PB (2009). Allosteric modulation of DNA by small molecules.. Proc Natl Acad Sci USA.

[9] Nguyen-Hackley DH, Ramm E, Taylor CM, Joung JK, Dervan PB (2004). Allosteric inhibition of zinc-finger binding in the major groove of DNA by minor-groove binding ligands.. Biochemistry.

[10] Fiamegos YC, Kastritis PL, Exarchou V, Han H, Bonvin AMJJ (2011). Antimicrobial and efflux pump inhibitory activity of caffeoylquinic acids from artemisia absinthium against gram-positive pathogenic bacteria.. PloS ONE.

[11] Muzikar KA, Nickols NG, Dervan PB (2009). Repression of DNA-binding dependent glucocorticoid receptor-mediated gene expression.. Proc Natl Acad Sci USA.

[12] van Tilborg MAA, Lefstin JA, Kruiskamp M, Teuben JM, Boelens R (2000). Mutations in the glucocorticoid receptor DNA-binding domain mimic an allosteric effect of DNA.. J Mol Biol.

[13] Goetz TL, Gu TL, Speck NA, Graves BJ (2000). Auto-inhibition of Ets-1 is counteracted by DNA binding cooperativity with core-binding factor α2.. Mol Cell Biol.

[14] Hsu CF, Dervan PB (2008). Quantitating the concentration of Py-Im polyamide-fluorescein conjugates in live cells.. Bioorg Med Chem Lett.

[15] Dervan PB, Edelson BS (2003). Recognition of the DNA minor groove by pyrrole-imidazole polyamides.. Curr Opin Struct Biol.

[16] Rogatsky I, Wang JC, Derynck MK, Nonaka DF, Khodabakhsh DB (2003). Target-specific utilization of transcriptional regulatory surfaces by the glucocorticoid receptor.. Proc Natl Acad Sci USA.

[17] van Tilborg MAA, Bonvin AMJJ, Hard K, Davis AL, Maler B (1995). Structure refinement of the glucocorticoid receptor-DNA binding domain from NMR data by relaxation matrix calculations.. J Mol Biol.

[18] Lonard DM, Lanz RB, O'Malley BW (2007). Nuclear receptor coregulators and human disease.. Endocr Rev.

[19] Luisi BF, Xu WX, Otwinowski Z, Freedman LP, Yamamoto KR (1991). Crystallographic analysis of the interaction of the glucocorticoid receptor with DNA.. Nature.

[20] Diamond MI, Robinson MR, Yamamoto KR (2000). Regulation of expanded polyglutamine protein aggregation and nuclear localization by the glucocorticoid receptor.. Proc Natl Acad Sci USA.

[21] Presman D, Alvarez LD, Levi V, Eduardo S, Digman MA (2010). Insights on glucocorticoid receptor activity modulation through the binding of rigid steroids.. PLoS One.

[22] Stockner T, Sterk H, Kaptein R, Bonvin AMJJ (2003). Molecular dynamics studies of a molecular switch in the glucocorticoid receptor.. J Mol Biol.

[23] Harris LF, Sullivan MR, Hickok DF (1993). Conservation of genetic information: A code for site-specific DNA recognition.. Proc Natl Acad Sci USA.

[24] Harris LF, Sullivan MR, Popken-Harris PD, Hickok DF (1995). A one nanosecond molecular dynamics simulation of the glucocorticoid receptor protein in complex with a glucocorticoid response element DNA sequence in a 10 Angstrom water layer.. J Biomol Struct Dyn.

[25] Bishop TC, Schulten K (1996). Moleculae dynamics study of glucocorticoid receptor-DNA binding.. Proteins: Struct Funct Genet.

[26] Leulliot N, Varani G (2003). Current topics in RNA-protein recognition: Control of specificity and biological function through induced fit and conformational capture.. Biochemistry.

[27] Zhang Q, Dwyer TJ, Tsui V, Case DA, Cho J (2004). NMR structure of a cyclic polyamide-DNA complex.. J Am Chem Soc.

[28] Rocha-Viegas L, Vicent GP, Barañao JL, Beato M, Pecci A (2006). Glucocorticoids repress bcl-X expression in lymphoid cells by recruiting STAT5B to the P4 promoter.. J Biol Chem.

[29] Huey R, Morris GM, Olson AJ, Goodsell DS (2007). A semiempirical free energy force field with charge-based desolvation.. J Comput Chem.

[30] Morris GM, Goodse DS, Halliday RS, Huey R, Hart WE (1998). Automated docking using a Lamarckian genetic algorithm and an empirical binding free energy function.. J Comput Chem.

[31] Sanner MF (1999). Python: A programming language for software integration and development.. J Mol Graph Model.

[32] Case DA, Darden TA, Cheatham TE, Simmerling CL, Wang JM (2006).

[33] Yang B, Zhu YY, Wang Y, Chen GJ (2011). Interaction identification of Zif268 and TATAZF proteins with GC-/AT-rich DNA sequence: a theoretical study.. J Comput Chem.

[34] Duan Y, Wu C, Chowdhury S, Lee MC, Xiong G (2003). A point-charge force field for molecular mechanics simulations of proteins based on condensed-phase quantum mechanical calculations.. J Comput Chem.

[35] Lee MC, Duan Y (2004). Distinguish protein decoys by using a scoring function based on a new AMBER force field, short molecular dynamics simulations, and the generalized born solvent model.. Proteins.

[36] Perez A, Marchan I, Svozil D, Sponer J, Cheatham TE (2007). Refinement of the AMBER force field for nucleic acids: improving the description of alpha/gamma conformers.. Biophys J.

[37] Wang J, Wolf RM, Caldwell JW, Kollamn PA, Case DA (2004). Development and testing of a general Amber force field.. J Comput Chem.

[38] Cheatham TE, Srinivasan J, Case DA, Kollman PA (1998). Molecular dynamics and continuum solvent studies of the stability of polyG-polyC and polyA-polyT DNA duplexes in solution.. J Biomol Stuct Dyn.

[39] Kollman PA, Massova I, Reyes C, Kuhn B, Huo S (2000). Calculating structures and free energies of complex molecules: combining molecular mechanics and continuum models.. Acc Chem Res.

[40] Jayaram B, Sprous D, Young MA, Beveridge DL (1998). Free energy analysis of the conformational preferences of A and B forms of DNA in solution.. J Am Chem Soc.

[41] Srinivasan J, Cheatham TE, Cieplak P, Kollman PA, Case DA (1998). Continuum solvent studies of the stability of DNA, RNA, and phosphoramidate DNA helices.. J Am Chem Soc.

[42] McQuarrie DA (1976). Statistical mechanics..

[43] Kottalam J, Case DA (1990). Langevin modes of macromolecules: Applications to crambin and DNA hexamers.. Biopolymers.

[44] Jensen F (1999). Introduction to computational chemistry..

[45] Lavery R, Sklenar H (1988). The definition of generalized helicoidal parameters and of axis curvature for irregular nucleic acids.. J Biol Struct Dyn.

[46] Strahs D, Schlick T (2000). A-tract bending: insights into experimental structures by computational models.. J Mol Biol.

[47] Neidle S (1994). DNA Structure and Recognition..

[48] Kosikov KM, Gorin AA, Lu XJ, Olson WK, Manning GS (2002). Bending of DNA by Asymmetric Charge Neutralization: All-Atom Energy Simulations.. J Am Chem Soc.

[49] Dervan PB (2001). Molecular recognition of DNA by small molecules.. Bioorg Med Chem.

[50] Kielkopf CL, White S, Szewczyk JW, Turner JM, Baird EE (1998). A structural basis for recognition of A·T and T·A base pairs in the minor groove of B-DNA.. Science.

[51] So AY, Chaivorapol C, Bolton EC, Li H, Yamamoto KR (2007). Determinants of cell- and gene-specific transcriptional regulation by the glucocorticoid receptor.. PLoS Genet.

[52] Pufall MA, Graves BJ (2002). Autoinhibitory domains: modular effectors of cellular regulation.. Annu Rev Cell Dev Biol.

[53] Lee GM, Pufall MA, Meeker CA, Kang HS, Graves BJ (2008). The affinity of Ets-1 for DNA is modulated by phosphorylation through transient interactions of an unstructured region.. J Mol Biol.

