# Supplementary figures and images for: Allosteric Analysis of Glucocorticoid Receptor-DNA Interface Induced by Cyclic Py-Im Polyamide: A Molecular Dynamics Simulation Study
Source: PLoS One. 2012 Apr 19;7(4):e35159. doi: 10.1371/journal.pone.0035159 (PMC3331974; doi:10.1371/journal.pone.0035159)

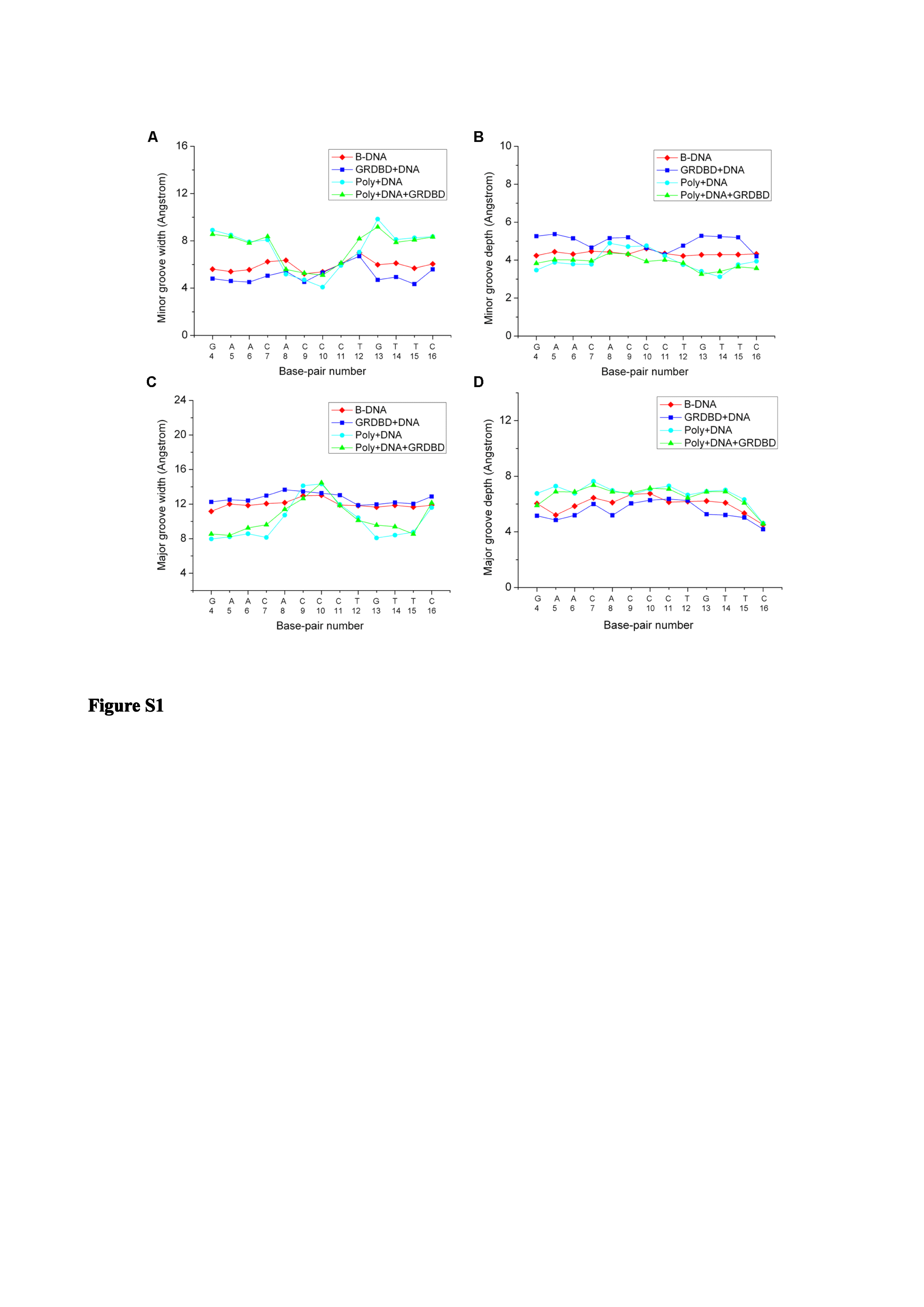

Supplement: Figure S1 — Groove widths and depths of B-DNA, GRDBD+DNA, Poly+DNA and Poly+DNA+GRDBD models. Minor groove widths (A), minor groove depths (B), major groove widths (C) and major groove depths (D) for the time-averaged structures of the DNA conformations in B-DNA (red line with diamond), GRDBD+DNA (blue line with square), Poly+DNA (cyan line with circle), and Poly+DNA+GRDBD (green line with up-triangle) models. (TIF) [file pone.0035159.s001.tif]

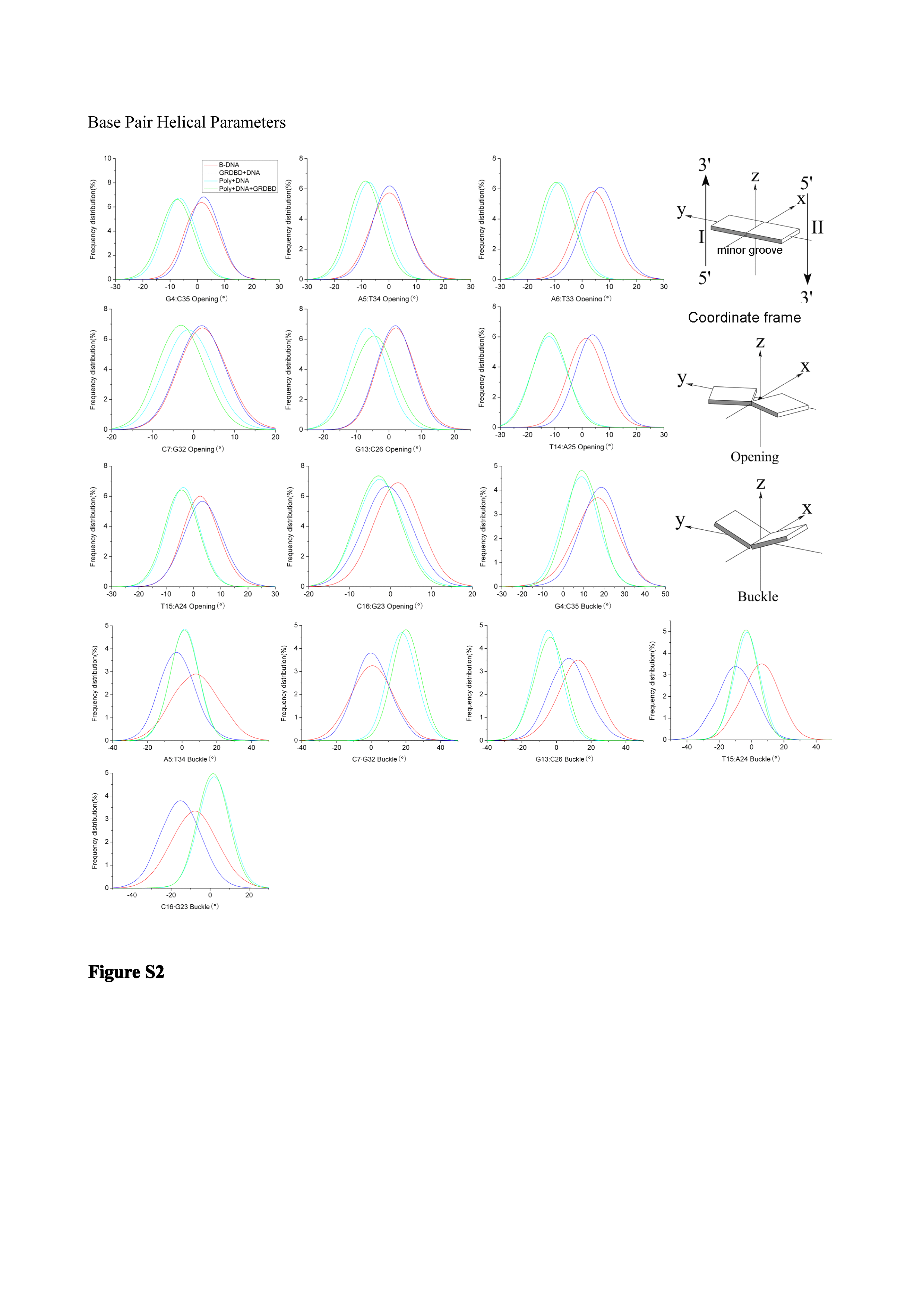

Supplement: Figure S2 — Frequency distributions of DNA base-pair helical parameters of B-DNA, GRDBD+DNA, Poly+DNA and Poly+DNA+GRDBD models. Selected frequency distributions of the representative DNA duplex base-pair helical parameters for the central binding base-pairs for B-DNA (red line), GRDBD+DNA (blue line), Poly+DNA (cyan line), and Poly+DNA+GRDBD (green line) models. (TIF) [file pone.0035159.s002.tif]

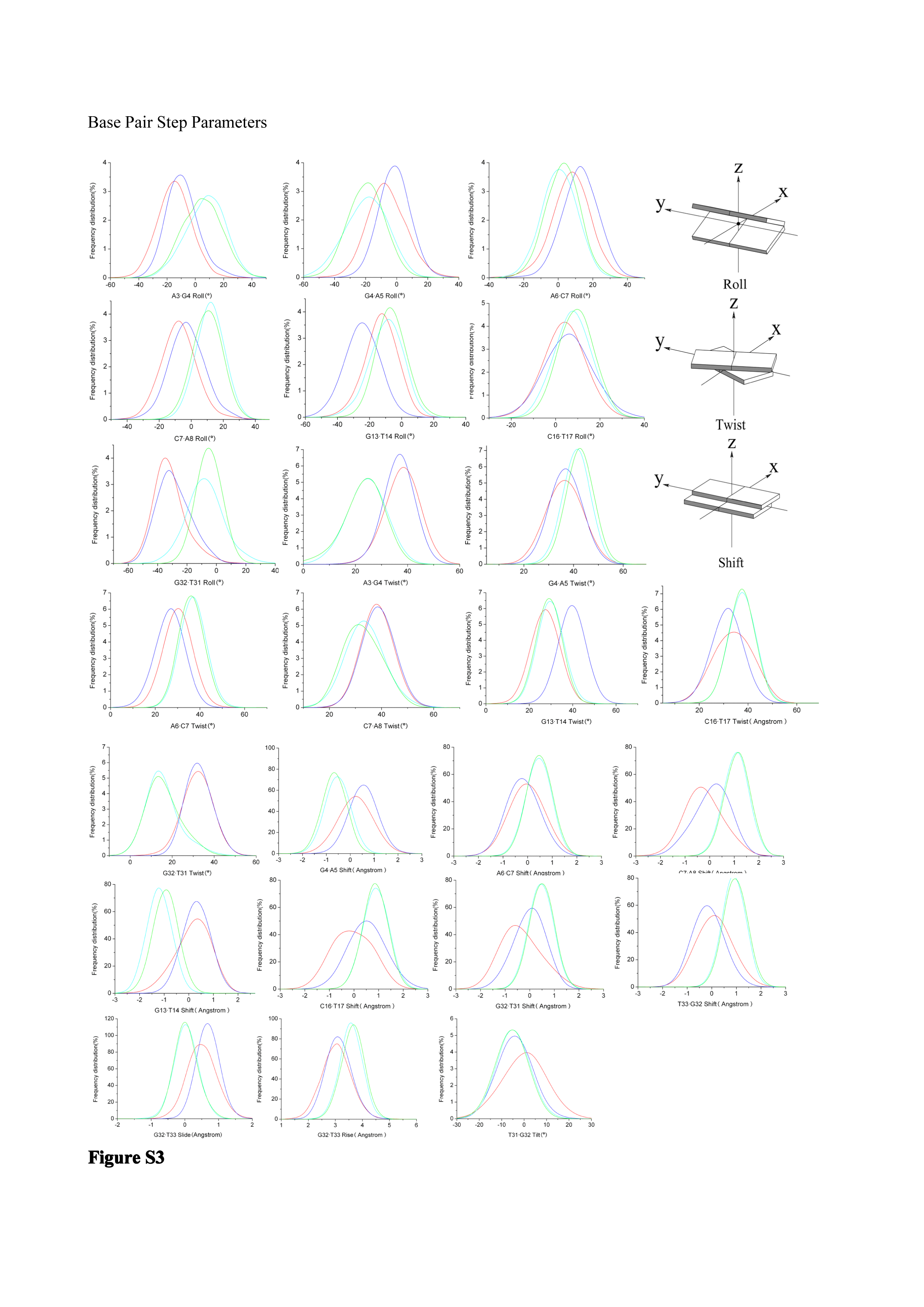

Supplement: Figure S3 — Frequency distributions of DNA base-pair step parameters of B-DNA, GRDBD+DNA, Poly+DNA and Poly+DNA+GRDBD models. Selected frequency distributions of the representative DNA duplex base-pair step parameters for the central binding base-pairs for B-DNA (red line), GRDBD+DNA (blue line), Poly+DNA (cyan line), and Poly+DNA+GRDBD (green line) models. (TIF) [file pone.0035159.s003.tif]

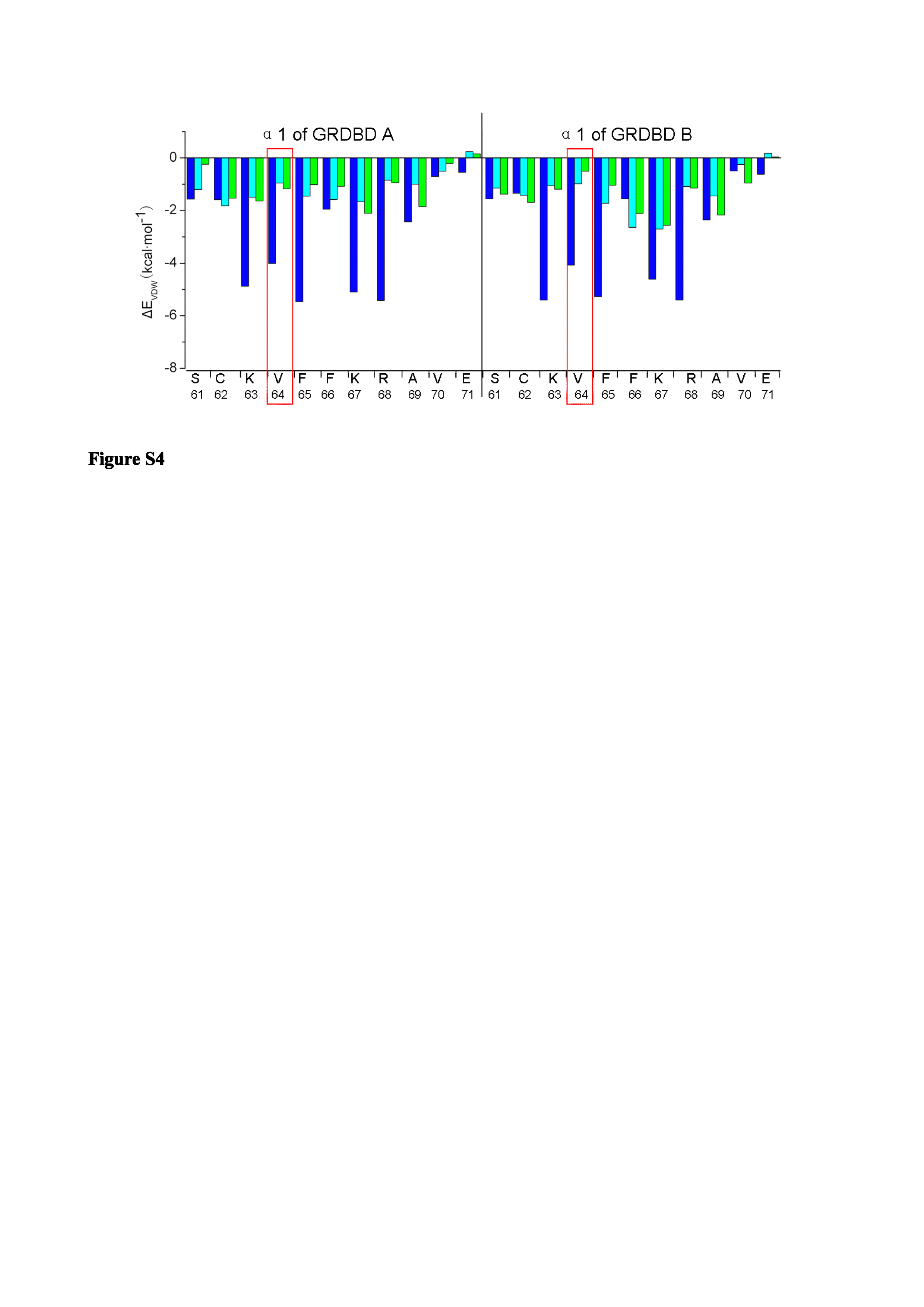

Supplement: Figure S4 — Energy decompositions of GRDBD dimer. MM-PBSA energy decompositions presented in the two α1 helical regions for the GRDBD dimer in kcal·mol−1: blue bars for the GRDBD+DNA model; cyan bars for the Poly+DNA+GRDBD model; green bars for the alloDNA+GRDBD model. (TIF) [file pone.0035159.s004.tif]

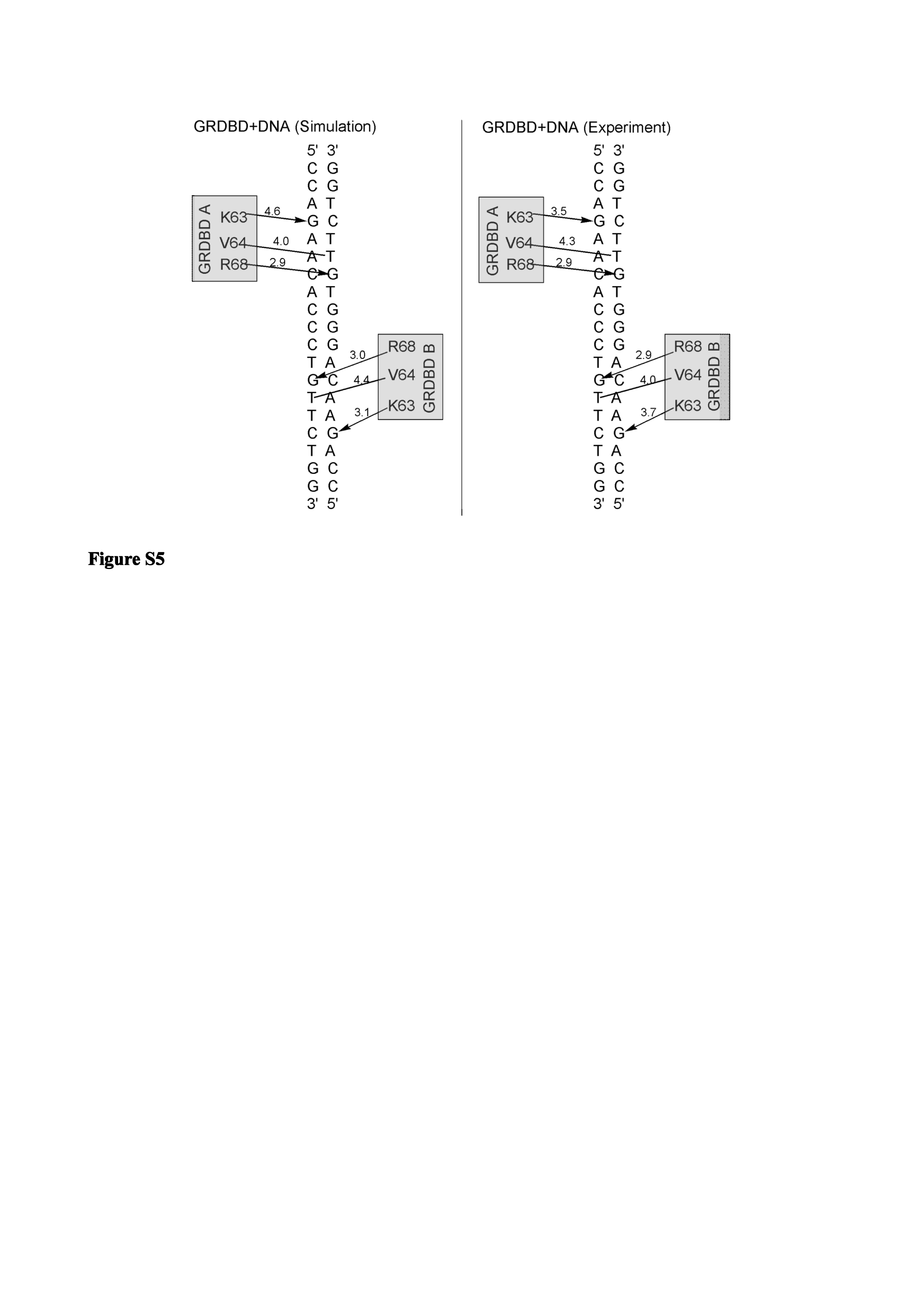

Supplement: Figure S5 — Contact sites and distances of GRDBD dimer with DNA. Diagrams of the calculated key contact sites and the corresponding distances (Å) of the GRDBD dimer with DNA for the simulated GRDBD+DNA model (left) compared with the experimental results (right). Hydrogen bond interactions indicated by black arrows and van der Waals interactions indicated by black line. (TIF) [file pone.0035159.s005.tif]

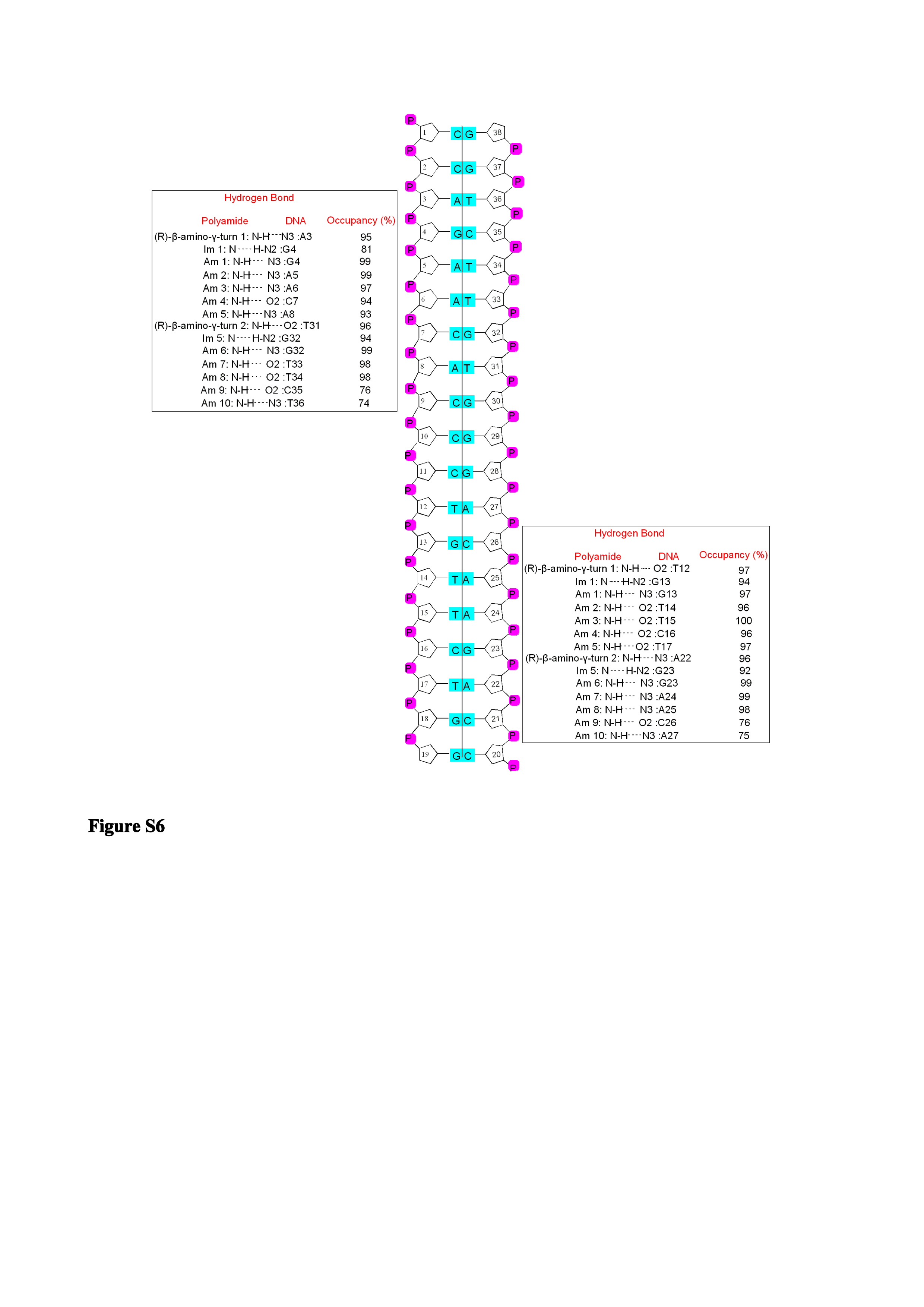

Supplement: Figure S6 — Hydrogen bonds between polyamides and DNA. Hydrogen bond map for two polyamides in the Poly+DNA and Poly+DNA+GRDBD models. (TIF) [file pone.0035159.s006.tif]

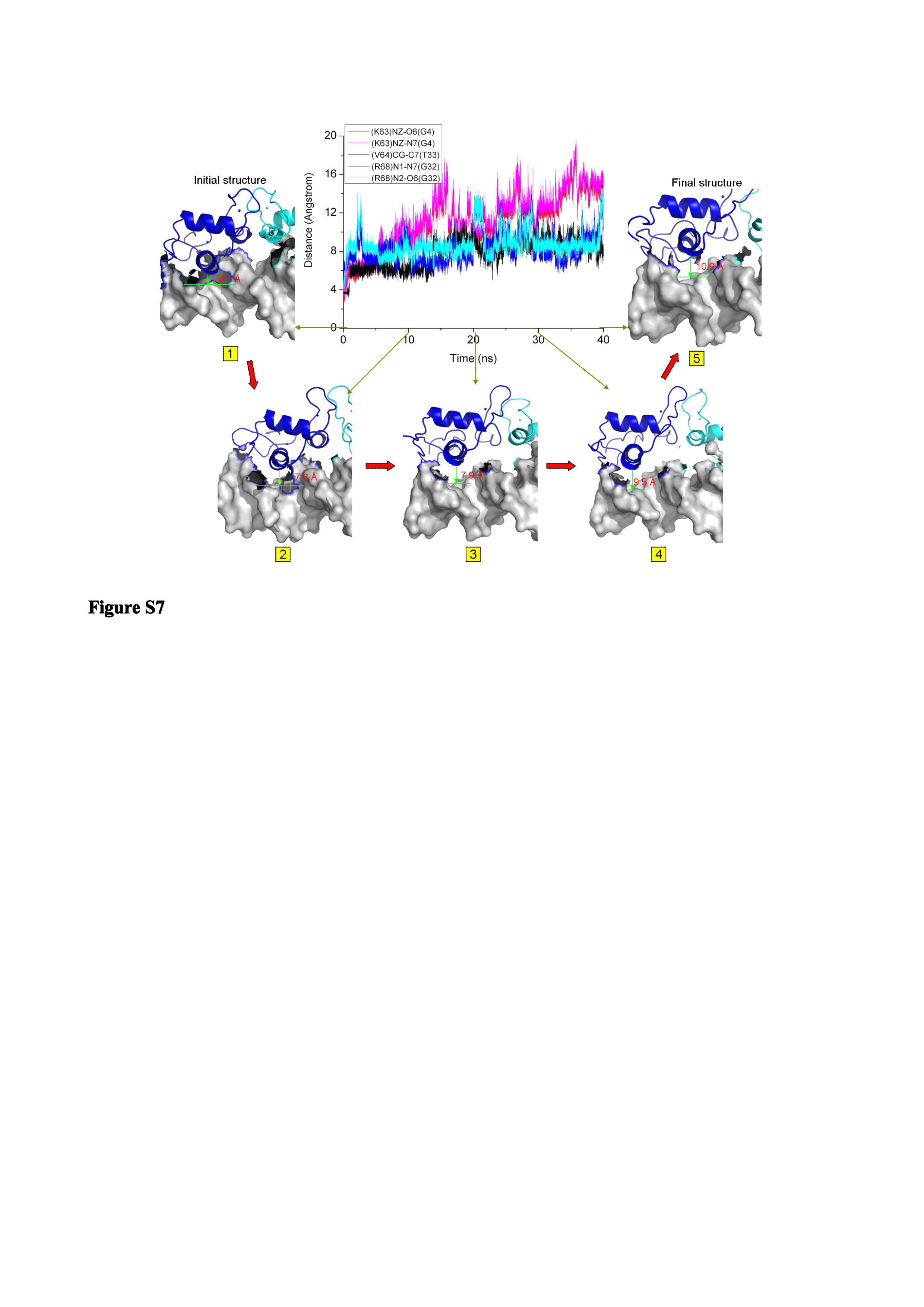

Supplement: Figure S7 — Distances at certain times for alloDNA+GRDBD model. The center image is the time-dependence of distances at the connection sites between the residues of GRDBD A and the bases of DNA during the simulation of the alloDNA+GRDBD model. Around the center image, five snapshots are extracted from the alloDNA+GRDBD trajectory at the times of 0 (1), 10 (2), 20 (3), 30 (4) and 40 (5) ns, respectively, along with the average distances of connection sites. (TIF) [file pone.0035159.s007.tif]

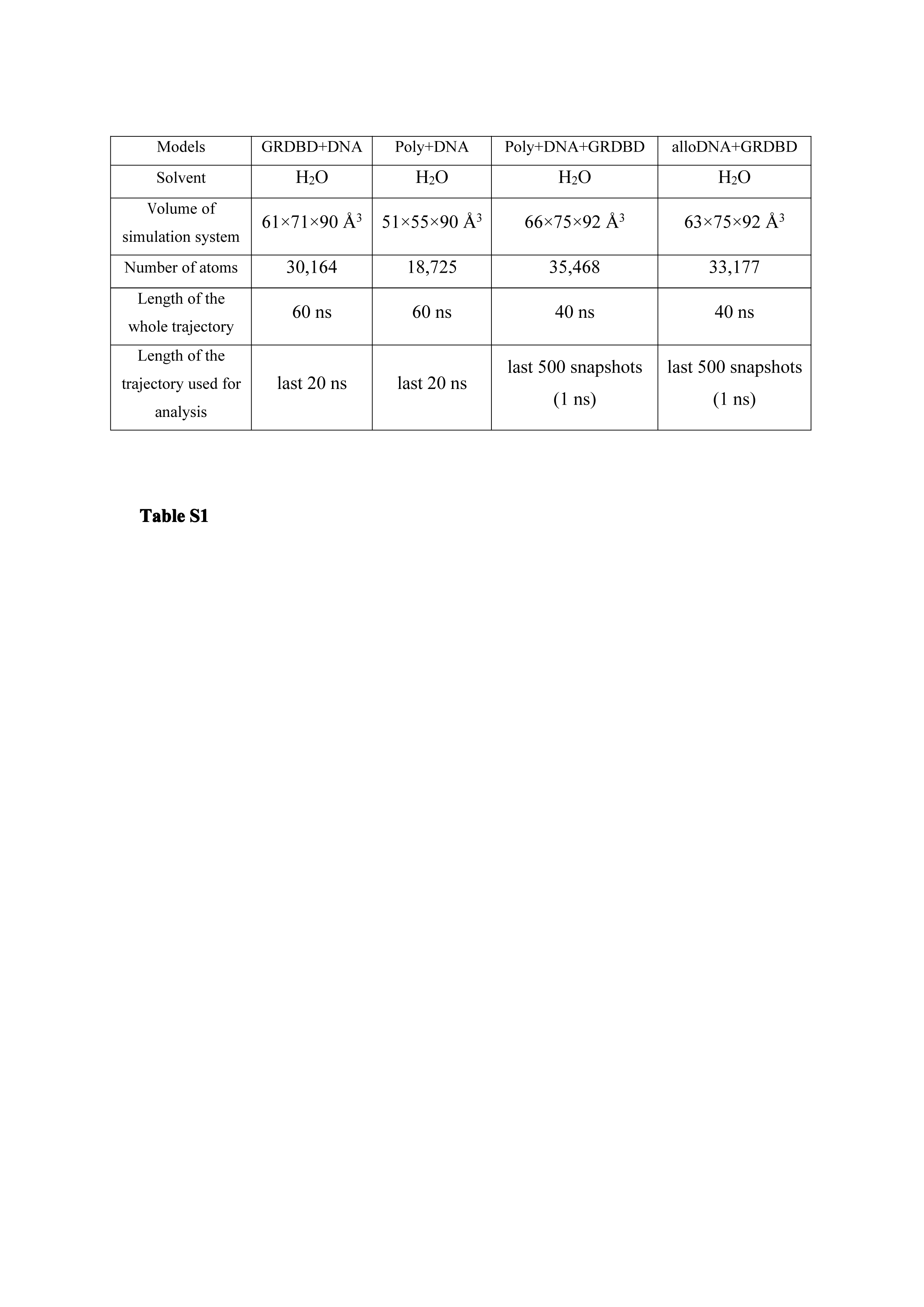

Supplement: Table S1 — Summary of the simulations performed in this work. (TIF) [file pone.0035159.s008.tif]

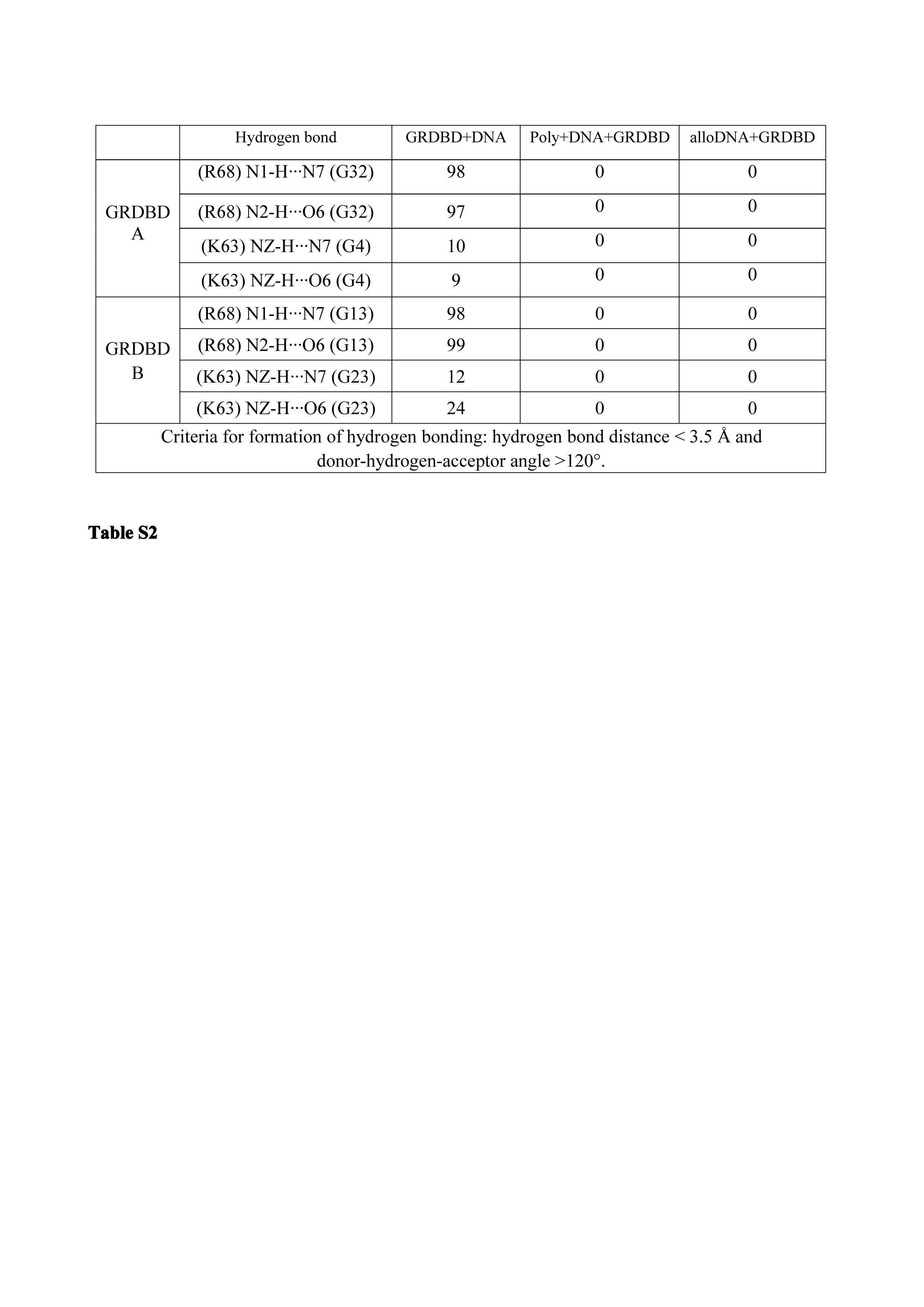

Supplement: Table S2 — The occupancy (%) of hydrogen bonds between the GRDBD dimer and DNA for the GRDBD+DNA, Poly+DNA+GRDBD, and alloDNA+GRDBD models. (TIF) [file pone.0035159.s009.tif]
