# Supplementary material for: Allosteric Analysis of Glucocorticoid Receptor-DNA Interface Induced by Cyclic Py-Im Polyamide: A Molecular Dynamics Simulation Study
Source: PLoS One. 2012 Apr 19;7(4):e35159. doi: 10.1371/journal.pone.0035159 (PMC3331974; doi:10.1371/journal.pone.0035159)
Supplement: Text S1 — Docking protocols for Poly+DNA model. (DOC) [file pone.0035159.s010.doc]

**Text S1**

**Docking protocols**

AutoDock 4.0 software package [1,2] was used for conducting docking studies on the Poly+DNA model. The graphical user interface program AutoDock Tools [3] was used to prepare, run, and analyze the docking simulations. The Kollman charges, solvation parameters and polar hydrogen were added into the water free DNA structure for the preparation of DNA in docking simulation. The Gasteiger charge was assigned into polyamide and then non-polar hydrogen was merged. The rigid root of polyamide was defined automatically instead of picking manually. AutoDock requires pre-calculated grid map in which each atom type in polyamide and its potential energy arising from the interaction with macromolecule have been presented. This grid must surround the region of interest in the macromolecule. AutoGrid 4.0 Program, supplied with AutoDock 4.0 was used to generate grid map for polyamide. The box size in x-, y- and z- axis was normally set at 50Å×40Å×60Å. The spacing between grid points was 0.375 Å. The GA-LS search algorithm (Lamarckian Genetic Algorithm) was chosen to search for the best conformers with 10 runs. During the searching process, both DNA and cyclic polyamide were regarded as rigid, to explore in any of the degrees of freedom. A default protocol was applied, with an initial population of 150 randomly placed individuals, a maximum number of 250,000 energy evaluations, and a maximum number of 27,000 generations. A mutation rate of 0.02 and a cross-over rate of 0.8 were used. The best docked conformation of polyamide into DNA binding sites was determined as the one which has the lowest interaction energies of both docking energy and binding free energy among the 10 different poses generated. Such conformation of this model has been selected as the initial structure for MD simulation.

**References**

1. Morris GM, Goodse DS, Halliday RS, Huey R, Hart WE, et al. (1998) Automated docking using a Lamarckian genetic algorithm and an empirical binding free energy function. J Comput Chem 19: 1639-1662.

2. Huey R, Morris GM, Olson AJ, Goodsell DS (2007) A semiempirical free energy force field with charge-based desolvation. J Comput Chem 28: 1145-1152.

3. Sanner MF (1999) Python: A programming language for software integration and development. J Mol Graph Model 17: 57-61.
