# Supplementary material for: Allosteric Analysis of Glucocorticoid Receptor-DNA Interface Induced by Cyclic Py-Im Polyamide: A Molecular Dynamics Simulation Study
Source: PLoS One. 2012 Apr 19;7(4):e35159. doi: 10.1371/journal.pone.0035159 (PMC3331974; doi:10.1371/journal.pone.0035159)
Supplement: Text S3 — MM-PBSA calculation for free energy. (DOC) [file pone.0035159.s012.doc]

**Text S3**

**MM-PBSA calculation for free energy**

Energetic post-process of single-trajectory/triplet-trajectory was performed for each MM-PBSA calculation by using the MM-PBSA module of AMBER9 program through molecular mechanics and a continuum solvent model [1]. In MM-PBSA calculation, *G*_np/solv_ is non-polar solvation free energy, which was calculated by using a solvent accessible surface area (SASA) as follows:

*G*_np/solv_ = rSASA + b

The SASA is the solvent-accessible surface area, and is estimated using Sanner’s algorithm implemented in the Molsurf program in AMBER9 [2] with a probe radius of 1.4 Å. The surface tension proportionality constant (r) and the free energy of non-polar solvation for a point solute (b) are set to 0.00542 kcal mol^-1^ Å^-2^ and 0.92 kcal mol^-1^, respectively.

For each of the GRDBD+DNA and Poly+DNA models, the last 20ns trajectory of the production dynamics stage was used for binding free energy calculations of MM-PBSA, namely, the 500 snapshots of each model at a 40-ps interval for computation of enthalpy and 20 snapshots at 1000-ps intervals for computation of entropy. For each of the Poly+DNA+GRDBD and alloDNA+GRDBD models, only the last 1ns trajectory, i.e. the last 500 snapshots of the corresponding trajectory at a 2-ps interval, was analyzed for computations of enthalpy, and only the last 20 snapshots at a 50-ps interval in each trajectory were selected for the entropy analyses. In addition, the MM-PBSA energy decompositions by per-residue for the four systems were performed to address the contribution of each residue to the binding free energies, which can provide a full description of energetic influences on binding affinity. Per-residue free energy decompositions were performed on the molecular mechanics, solvation free energies that were calculated by the generalized Born method, and surface-based energies. As Poisson-Boltzmann energies cannot be decomposed by residues, only results obtained with the generalized Born method are reported in this work [3].

**References**

1. Case DA, Darden TA, Cheatham TE III, Simmerling CL, Wang JM, et al. (2006) University of California, San Francisco.

2. Connolly ML (1983) Analytical molecular surface calculation. J Appl Cryst 16: 548-558.

3. Gohlke H, Kiel C, Case DA (2003) Insights into protein–protein binding by binding free energy calculation and free energy decomposition for the Ras–Raf and Ras–RalGDS complexes. J Molec Biol 330: 891-913.
