# Supplementary material for: Allosteric Analysis of Glucocorticoid Receptor-DNA Interface Induced by Cyclic Py-Im Polyamide: A Molecular Dynamics Simulation Study
Source: PLoS One. 2012 Apr 19;7(4):e35159. doi: 10.1371/journal.pone.0035159 (PMC3331974; doi:10.1371/journal.pone.0035159)
Supplement: Text S4 — DNA helical parameter analysis of trajectories. (DOC) [file pone.0035159.s013.doc]

**Text S4**

**DNA helical parameter analysis of trajectories**

The PTRAJ module of AMBER9 program was used to extract the production conformations. These extracted snapshots were saved in the Protein Data Bank (PDB) format. Each nucleotide type was converted from AMBER format to PDB format, and the resulting snapshots were submitted to CURVES program [1]. The following CURVES parameters were extracted, i.e., global base-pair helical parameters: shear, stretch, stagger, buckle, propeller and opening; and global inter base-pair step helical parameters: shift, slide, rise, tilt, roll and twist. Percentage occupancy distributions of the DNA helical parameters were calculated by normalizing the frequency distributions to 100%. Moreover, the overall bend of DNA for the time-averaged structures of the GRDBD+DNA, Poly+DNA, and Poly+DNA+GRDBD models were calculated from the CURVES output using MadBend program (http:// modod.biomath.nyu.edu) developed by Strahs and Schlick [2]. This method evaluates the DNA curvature by summing the projected components of local base pair step tilt and roll angles after adjusting the helical twist. Bend in the helical axis defined by a negative roll angle indicates bending toward the minor groove, while bend defined by a positive roll angle corresponds to bending toward the major groove [2].

**References**

1. Lavery R, Sklenar H (1988) The definition of generalized helicoidal parameters and of axis curvature for irregular nucleic acids. J Biol Struct Dyn 6: 63-91.

2. Strahs D, Schlick T (2000) A-tract bending: insights into experimental structures by computational models. J Mol Biol 301: 643-663.
